# Case Report: Discordant diffuse large B-cell lymphoma and splenic peripheral T-cell lymphoma revealed by persistent splenomegaly and cytopenia

**DOI:** 10.3389/fonc.2026.1897274

**Published:** 2026-07-20

**Authors:** Zhengying Ruan, Guangmin Fan, Linglong Xu

**Affiliations:** 1 Department of Pathology, Taizhou Central Hospital (Taizhou University Hospital), Taizhou, China; 2 Department of Hematology, Taizhou Central Hospital (Taizhou University Hospital), Taizhou, China

**Keywords:** case report, composite lymphoma, cytopenia, diffuse large B-cell lymphoma, discordant lymphoma, peripheral T-cell lymphoma, spleen, splenomegaly

## Abstract

Discordant lymphomas involving mature B-cell and T-cell neoplasms are uncommon and may be missed when atypical clinical manifestations are attributed to a known aggressive lymphoma. We report a 71-year-old man who presented with fever, generalized lymphadenopathy, splenomegaly, splenic infarction, anemia, and thrombocytopenia. Bone marrow, flow cytometry, and tissue immunohistochemistry supported stage IVB diffuse large B-cell lymphoma (DLBCL), non-germinal-center B-cell-like subtype, with bone marrow involvement. After dose-reduced R-CHOPE chemotherapy, partial remission was achieved, but thrombocytopenia and marked splenomegaly persisted. Splenectomy, performed for suspected hypersplenism, unexpectedly showed peripheral T-cell lymphoma with loss of B-cell markers and clonal TCRG and TCRB rearrangements. Ten months after presentation, a right zygomatic-orbital soft tissue lesion was biopsied and confirmed recurrent DLBCL. The patient received R-GemOx plus zanubrutinib, intrathecal chemotherapy, and later R-ICE plus zanubrutinib; however, repeated relapse occurred, and he died 33 months after initial presentation. This case illustrates that persistent splenomegaly, cytopenia, splenic infarction, or anatomically discordant progression during lymphoma treatment should trigger repeat tissue sampling and integrated pathological reassessment. The distinct anatomical distribution, lineage-specific immunophenotypes, and splenic T-cell clonality supported the diagnosis of lineage-discordant lymphoma.

## Introduction

Composite and discordant lymphomas are uncommon diagnostic entities in which more than one lymphoma subtype is identified in a single patient. Composite lymphoma usually refers to two morphologically and immunophenotypically distinct lymphomas in the same anatomical site, whereas discordant lymphoma is used when different histologies are detected at separate sites. Population-based data indicate that sequential or composite lymphoma represents only a small fraction of lymphoma diagnoses (1). Combinations involving B-cell and T-cell lineages are particularly rare, but they are clinically important because the second component may alter diagnostic interpretation, staging, and treatment planning.

Published reports show several recurring patterns rather than a single clinicopathological entity. One reported pattern is DLBCL coexisting with PTCL in nodal or systemic disease (2). Cutaneous or subcutaneous presentations have also been described, including DLBCL-leg type with subcutaneous panniculitis-like T-cell lymphoma (3). Other reports involve intestinal or broader gastrointestinal presentations (4, 5). EBV-positive large B-cell proliferation in primary cutaneous PTCL represents another cutaneous B/T-cell composite pattern (6). Sequential or composite disease involving angioimmunoblastic T-cell lymphoma or extranodal NK/T-cell lymphoma together with DLBCL has also been reported (7–9).

Splenic-predominant PTCL is particularly uncommon. Splenic involvement by peripheral T-cell and NK-cell neoplasms shows heterogeneous pathological patterns, and the literature on splenic T/NK-cell lymphoma is limited compared with that on B-cell lymphoma (10). Only isolated reports have described primary or predominant splenic localization of PTCL-NOS (11). Therefore, persistent splenomegaly, splenic infarction, or cytopenia in a patient with DLBCL may create a diagnostic pitfall because residual spleen-related findings can be attributed to the known B-cell lymphoma, infection, or hypersplenism rather than a second lymphoma component.

Many published reports have emphasized molecular or viral associations, especially Epstein-Barr virus (EBV)-driven B-cell proliferation in the setting of T-cell lymphomas or immune dysregulation (12–14). However, EBV-negative disease has also been reported, and some patients lack a clear immunosuppressive background, leaving the pathogenetic relationship uncertain (15).

We report a patient with nodal, marrow, and later orbital DLBCL, together with splenic PTCL discovered only after splenectomy for persistent splenomegaly and cytopenia. This case highlights a practical diagnostic problem in an unusual lymphoma presentation: persistent or anatomically discordant disease manifestations should not be assumed to represent the initially diagnosed lymphoma subtype.

## Case presentation

A 71-year-old man with hypertension was admitted in October 2021 after 10 days of fever, abdominal pain, cough, chest tightness, and multiple enlarged lymph nodes. Physical examination revealed bilateral supraclavicular, axillary, and inguinal lymphadenopathy, mild abdominal distension, and bilateral lower-extremity edema. Laboratory tests showed systemic inflammation, anemia, thrombocytopenia, hypoalbuminemia, hyperferritinemia, and coagulation abnormalities: C-reactive protein 106.3 mg/L, procalcitonin 12.0 ng/mL, ferritin 1423.78 ng/mL, D-dimer 1.94 mg/L, albumin 23.3 g/L, hemoglobin 82 g/L, and platelet count 38 × 10^9/L. The karyotype was 46,XY. Molecular testing detected MYD88 L265P, with a reported mutation frequency of 46.10%. CD79B mutation testing was not performed. Ultrasonography showed marked splenomegaly with heterogeneous echogenicity and focal hypoechoic areas. Abdominal computed tomography angiography showed splenomegaly, pulmonary infection, and splenic infarction (**Figure 1**).

**Figure 1:**
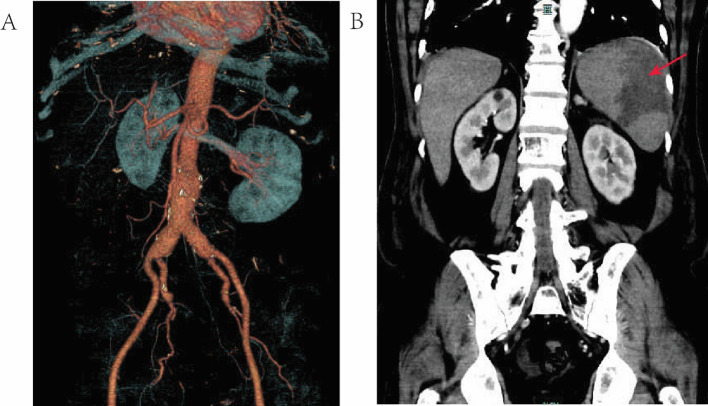
Imaging findings at presentation. **(A)** Abdominal computed tomography angiography showing the abdominal vasculature and splenomegaly. **(B)** Coronal computed tomography image showing splenomegaly and splenic infarction, indicated by the arrow.

Bone marrow smear and biopsy showed abnormal B-cell lymphoid proliferation with marrow infiltration. Lymphoma cells accounted for 49.5% of nucleated cells and showed variable cell size, irregular nuclear contours, cluster formation, and macrophage hemophagocytosis. Flow cytometry identified abnormal monoclonal B lymphocytes expressing CD5, CD19, CD20, and CD79α, lacking CD10, and showing restricted surface kappa light-chain expression. Immunohistochemistry showed CD20- and CD79α-positive large B cells with aberrant CD5 expression, while CD3 mainly highlighted admixed background T cells. Tumor cells expressed leukocyte common antigen, MUM1, BCL2, cyclin D1, and MYC in approximately 40% of cells; Ki-67 was approximately 80%. CD10, CD30, cytokeratin, BCL6, and EBV-encoded RNA were negative. These findings supported DLBCL, non-germinal-center B-cell-like subtype, stage IVB with bone marrow involvement (**Figure 2**).

**Figure 2:**
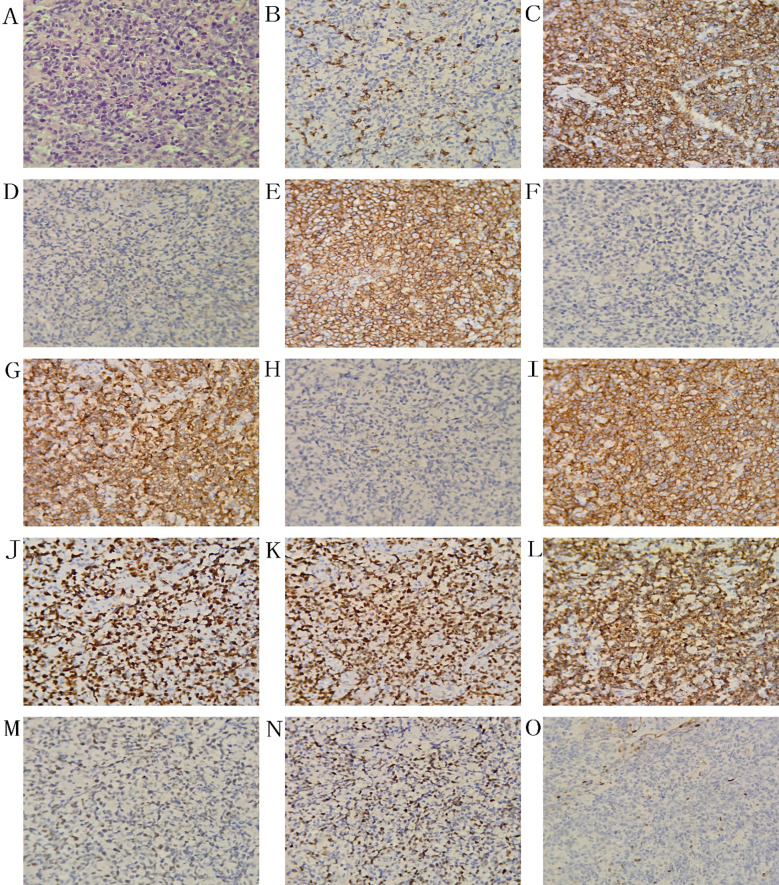
Histopathological and immunohistochemical findings in the lymph node. **(A)** Hematoxylin and eosin staining showing diffuse proliferation of atypical lymphoid cells. Immunohistochemistry showed CD3 highlighting admixed background T cells **(B)**, with positivity for CD5 **(C)**, CD20 **(E)**, CD79α **(G)**, leukocyte common antigen **(I)**, Ki-67 **(J)**, MUM1 **(K)**, BCL2 **(L)**, MYC **(N)**, and cyclin D1 **(O)**, and negativity for CD10 **(D)**, CD30 **(F)**, cytokeratin **(H)**, and BCL6 **(M)**. Original magnification, ×400.

After antimicrobial treatment, the fever resolved. From the second week after admission, the patient received two cycles of dose-reduced R-CHOPE, including rituximab, cyclophosphamide, doxorubicin, vincristine, dexamethasone, and etoposide, and achieved partial remission. During the second month after diagnosis, thrombocytopenia persisted despite supportive treatment, and marked splenomegaly remained. Because hypersplenism was suspected, splenectomy was performed.

## Splenic pathological reassessment

The splenectomy specimen showed loss of normal splenic architecture, diffuse proliferation of small lymphocytes, and areas of infarction. The lymphoid cells had irregular or twisted nuclear contours, small nucleoli, and scant cytoplasm, with an increased interstitial network of high endothelial venules. Immunohistochemistry showed positivity for CD3, CD4, CD5, CD8, CD31, CD43, CD56, CD68, TIA-1, and Ki-67 at approximately 20%. CD10, CD20, CD30, CD79α, CD99, CD117, granzyme B, epithelial membrane antigen, MUM1, BCL6, MYC, cyclin D1, myeloperoxidase, terminal deoxynucleotidyl transferase, EBV, and EBV-encoded RNA were negative. Vascular structures expressed ERG and CD34. Clonal rearrangements of TCRG and TCRB were detected in the splenic tissue. These findings supported a diagnosis of PTCL involving the spleen rather than persistent splenic involvement by the previously diagnosed DLBCL (**Figure 3**). The splenic lesion was therefore interpreted as PTCL involving the spleen, rather than being assigned to a more specific T/NK-cell lymphoma subtype. The expression of CD56 and TIA-1 suggested a cytotoxic or NK-associated phenotype; however, the absence of EBV/EBER expression, the absence of granzyme B, and the presence of clonal TCRG and TCRB rearrangements did not support a diagnosis of typical extranodal NK/T-cell lymphoma. The simultaneous expression of CD4 and CD8 was considered an aberrant mature T-cell immunophenotype. Although this finding is unusual, it further supported a neoplastic T-cell population when interpreted together with the abnormal splenic architecture and T-cell clonality.

**Figure 3:**
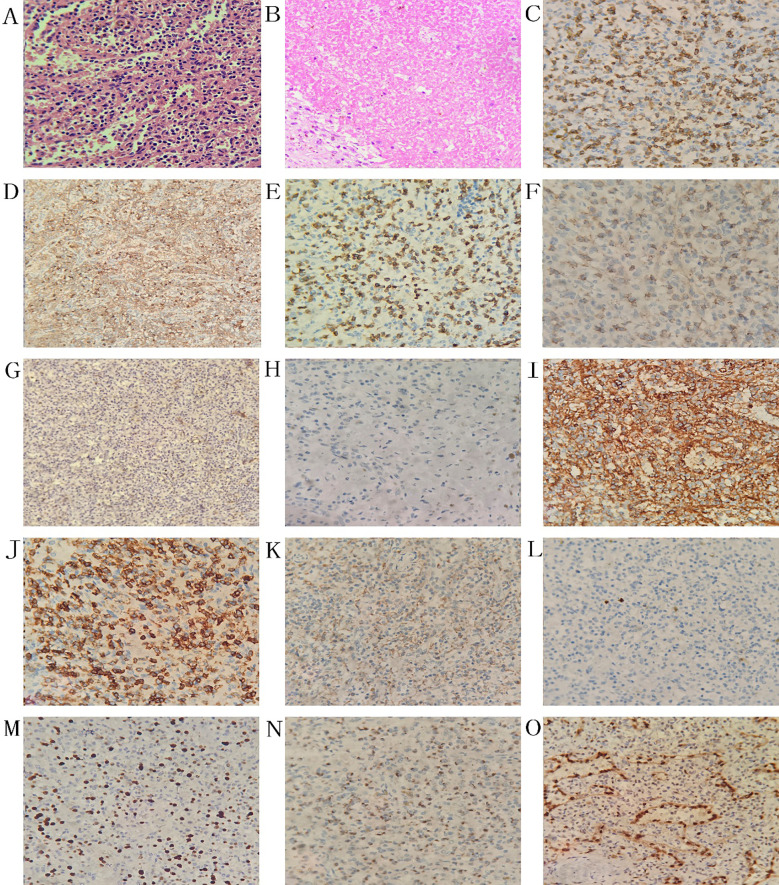
Histopathological and immunohistochemical findings in the spleen. **(A)** Hematoxylin and eosin staining showing loss of normal splenic architecture with diffuse proliferation of small lymphocytes. **(B)** Hematoxylin and eosin staining showing splenic infarction. Immunohistochemistry showed positivity for CD3 **(C)**, CD4 **(D)**, CD5 **(E)**, CD8 **(F)**, CD31 **(I)**, CD43 **(J)**, CD68 **(K)**, Ki-67 **(M)**, TIA-1 **(N)**, and ERG **(O)**, and negativity for CD10 **(G)**, CD20 **(H)**, and CD79α **(L)**. Original magnification, ×400.

## Relapse, treatment, and outcome

Ten months after presentation, the patient developed right orbital pain and swelling. A right zygomatic-orbital soft tissue mass was biopsied. Histology and immunohistochemistry confirmed recurrent DLBCL (**Figure 4**), with positivity for CD20, CD79α, PAX5, MUM1, BCL2, BCL6, MYC, p53, Ki-67, and aberrant CD5 expression; CD3 highlighted admixed background T cells. CD10, CD30, cytokeratin, and cyclin D1 were negative. Repeat bone marrow examination also confirmed DLBCL involvement. Positron-emission tomography-computed tomography showed abnormal glucose metabolism in the right zygomatic region, multiple lymph node regions, and suspected left mandibular involvement.

**Figure 4:**
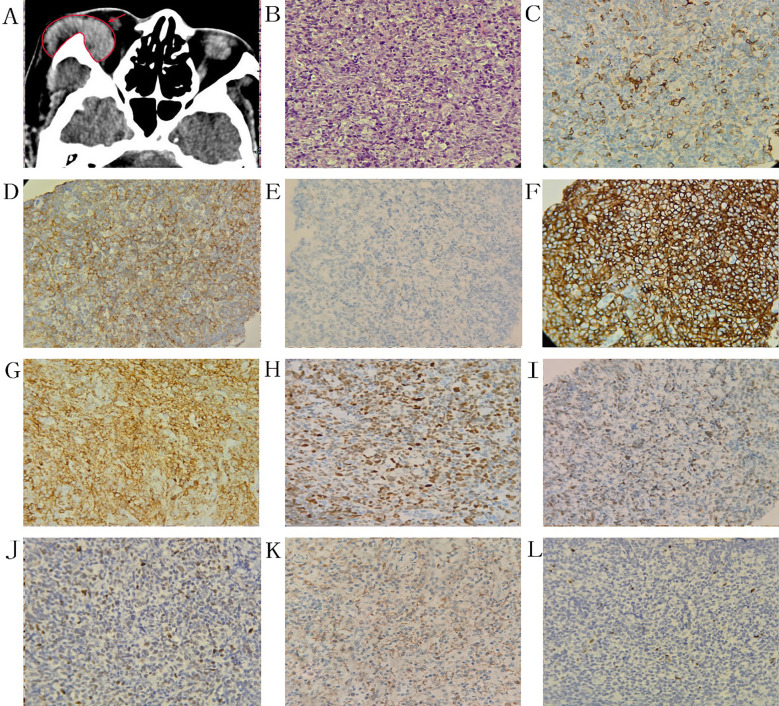
Imaging and pathological findings of the right zygomatic-orbital soft tissue lesion. **(A)** Computed tomography image showing a right zygomatic-orbital soft tissue mass without obvious bone destruction. **(B)** Hematoxylin and eosin staining showing diffuse proliferation of atypical lymphoid cells with enlarged nuclei, abnormal nuclear morphology, and visible mitotic figures. Immunohistochemistry showed CD3 highlighting admixed background T cells **(C)**, with positivity for CD5 **(D)**, CD20 **(F)**, CD79α **(G)**, Ki-67 **(H)**, BCL6 **(I)**, p53 **(J)**, and MUM1 **(K)**, and negativity for CD10 **(E)** and cyclin D1 **(L)**. Original magnification, ×400.

Treatment was changed to six cycles of R-GemOx plus zanubrutinib, consisting of rituximab, gemcitabine, oxaliplatin, and zanubrutinib. The right orbital mass nearly resolved, and zanubrutinib was continued as maintenance therapy. Intrathecal cytarabine, methotrexate, and dexamethasone were also administered. At 25 months, bilateral cervical lymphadenopathy recurred, accompanied by night sweats, abdominal distension, and lumbosacral discomfort. R-ICE plus zanubrutinib, including rituximab, ifosfamide, carboplatin, and etoposide, initially reduced the cervical nodes, but progressive neck lymphadenopathy and pain developed at 31 months. The patient died 33 months after initial presentation.

To improve chronological clarity in line with the CARE case report guidance, the overall clinical course, including diagnosis, treatment interventions, relapse events, and outcome, is summarized in **Figure 5** (16).

**Figure 5:**
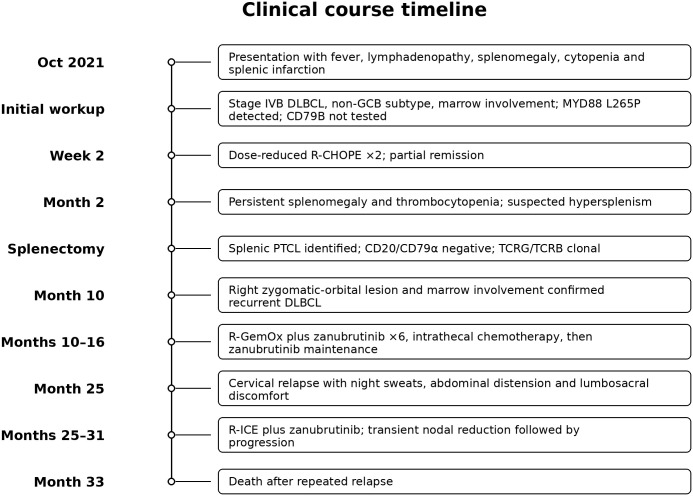
Clinical timeline of diagnosis, treatment, relapse, and outcome. The timeline summarizes the patient’s clinical course from initial presentation in October 2021 to death 33 months after presentation, including the initial diagnosis of DLBCL, dose-reduced R-CHOPE, persistent splenomegaly and thrombocytopenia, splenectomy revealing splenic PTCL, zygomatic-orbital relapse of DLBCL, R-GemOx plus zanubrutinib, intrathecal chemotherapy, subsequent R-ICE plus zanubrutinib, disease progression, and final outcome.

## Discussion

This case highlights a rare and clinically important form of lineage-discordant lymphoma. The initial disease was supported as DLBCL by marrow and tissue morphology, B-cell immunophenotype, flow-cytometric monoclonality, and later recurrent DLBCL in the zygomatic-orbital soft tissue. In contrast, the spleen showed a T-cell immunophenotype, absence of B-cell markers, and clonal TCRG/TCRB rearrangements. Because the B-cell and T-cell components were identified predominantly in different anatomical sites, the term discordant lymphoma is appropriate, although the broader literature on composite and sequential B-cell/T-cell lymphomas is also relevant (1–9).

Subclassification of the splenic T-cell lymphoma was challenging. Splenic involvement by peripheral T-cell and NK-cell neoplasms is uncommon, and the published pathological literature remains limited compared with B-cell lymphomas (10). In this case, the splenic tumor showed a mature T-cell phenotype with CD3, CD4, CD5, CD8, CD43, CD56, and TIA-1 expression, absence of B-cell markers, and clonal TCRG/TCRB rearrangements. CD56 and TIA-1 raised the possibility of an NK/T-cell or cytotoxic T-cell lineage (17). However, typical extranodal NK/T-cell lymphoma was not favored because EBV and EBER were negative, granzyme B was negative, and T-cell receptor clonality was present. Hepatosplenic T-cell lymphoma was also not favored because the available pathological and immunophenotypic findings did not show a typical hepatosplenic gamma-delta T-cell lymphoma pattern. T-cell large granular lymphocytic leukemia was considered less likely because the case lacked a documented chronic peripheral-blood LGL expansion and the presentation was dominated by destructive splenic tissue involvement. We therefore retained the conservative diagnosis of PTCL involving the spleen, with cytotoxic/NK-associated marker expression, rather than forcing the lesion into a more specific subtype.

The principal lesson from this case is diagnostic rather than mechanistic. At presentation, splenomegaly, splenic infarction, and thrombocytopenia could plausibly have been interpreted as manifestations of advanced DLBCL, infection, hypersplenism, or treatment-related complications. However, persistence of splenomegaly and cytopenia after chemotherapy led to splenectomy and revealed a second lymphoma component. This sequence supports a practical point for hematologists and pathologists: persistent or anatomically discordant disease manifestations during treatment should prompt repeat tissue sampling when feasible, particularly when the clinical course does not fully match the expected behavior of the established lymphoma subtype.

The pathogenesis of concurrent or sequential B-cell and T-cell lymphomas remains heterogeneous. Some cases, especially those associated with angioimmunoblastic T-cell lymphoma, show EBV-positive B-cell proliferation or arise in an immune-dysregulated microenvironment (12–14). Iatrogenic immune perturbation, including methotrexate exposure in cutaneous T-cell lymphoma, has also been associated with EBV-positive or related large B-cell lymphoproliferation (18, 19). Other reported cases are EBV negative or lack a clear immunosuppressive background, suggesting that independent clonal evolution may also occur (7, 15). In the present patient, EBV and EBV-encoded RNA were negative in the tested lesions, and there was no history of transplantation or long-term immunosuppression. Age-related immune dysfunction, host susceptibility, or treatment-related selective pressure may have contributed, but these possibilities remain inferential.

The MYD88 L265P mutation in this patient also requires careful interpretation. MYD88 L265P is classically associated with lymphoplasmacytic lymphoma/Waldenström macroglobulinemia and is also a defining alteration in the MCD genetic subtype of DLBCL when it co-occurs with CD79B mutation (20, 21). In the present case, the clinical presentation, marrow and lymph-node morphology, flow-cytometric monoclonal B-cell population, CD20/CD79α-positive immunophenotype, and later zygomatic-orbital relapse supported DLBCL rather than lymphoplasmacytic lymphoma. However, CD79B mutation testing was not performed. Therefore, although MYD88 L265P may suggest an activated B-cell-like or MCD-like molecular background, the DLBCL component could not be fully assigned to the MYD88/CD79B-mutated MCD genotype.

Treatment was individualized according to the clinically dominant and progressive component, as well as the patient’s age, performance status, and disease distribution. The initial use of dose-reduced R-CHOPE was consistent with the general principle that CD20-positive DLBCL is treated with rituximab-containing anthracycline-based chemoimmunotherapy (22), while dose adjustment reflected the patient’s age, inflammatory status, cytopenia, infection, and splenic infarction at presentation. After the zygomatic-orbital relapse confirmed DLBCL rather than splenic PTCL as the clinically progressive component, the treatment strategy shifted to salvage B-cell lymphoma therapy. R-GemOx was a reasonable less intensive salvage option because rituximab, gemcitabine, and oxaliplatin have shown activity and acceptable tolerability in relapsed or refractory DLBCL patients who are not candidates for high-dose therapy (23). R-ICE was also consistent with established salvage approaches for relapsed or primary refractory DLBCL (24). Zanubrutinib was added as an individualized targeted component rather than as a standard therapy; available evidence suggests only modest activity of zanubrutinib in relapsed or refractory non-GCB DLBCL, with possible greater relevance in tumors with BCR/NF-kappa B pathway alterations such as MYD88 and CD79B co-mutation (25). Because CD79B mutation was not tested in this case, the rationale for zanubrutinib should be considered exploratory. Therefore, the case supports individualized treatment guided by the dominant biopsy-proven component, but it does not establish a therapeutic standard for discordant B-cell/T-cell lymphoma.

## Conclusion

Persistent splenomegaly, thrombocytopenia, splenic infarction, discordant treatment response, or new extranodal disease in a patient with lymphoma should prompt repeat biopsy or tissue-based reassessment whenever feasible. In this patient, splenectomy revealed PTCL during treatment for DLBCL, changing the diagnostic interpretation from a single aggressive B-cell lymphoma to discordant B-cell and T-cell lymphoma. Integrated morphology, immunohistochemistry, flow cytometry, and TCR clonality testing provided the basis for diagnosis and highlighted a preventable diagnostic pitfall in rare hematologic malignancies.

## Patient perspective

The patient perspective was not available because the patient died during follow-up.

## Data Availability

The original contributions presented in the study are included in the article/supplementary material. Further inquiries can be directed to the corresponding authors.
